# Applications of molecular neuro-oncology - a review of diffuse glioma integrated diagnosis and emerging molecular entities

**DOI:** 10.1186/s13000-019-0802-8

**Published:** 2019-04-09

**Authors:** Matthew D. Wood, Aaron M. Halfpenny, Stephen R. Moore

**Affiliations:** 1 OHSU Department of Pathology, Division of Anatomic Pathology, Section of Neuropathology, Oregon Health & Science University, 3181 SW Sam Jackson Park Road, L-113, Portland, OR 97213 USA; 2 Knight Diagnostic Laboratories and Department of Molecular and Medical Genetics, Oregon Health & Science University, Portland, OR 97239 USA

**Keywords:** Brain tumors, Molecular pathology, Diffuse glioma, Integrated diagnosis, WHO 2016

## Abstract

Insights into the molecular underpinnings of primary central nervous system tumors have radically changed the approach to tumor diagnosis and classification. Diagnostic emphasis has shifted from the morphology of a tumor under the microscope to an integrated approach based on morphologic and molecular features, including gene mutations, chromosomal copy number alterations, and gene rearrangements. In 2016, the World Health Organization provided guidelines for making an integrated diagnosis that incorporates both morphologic and molecular features in a subset of brain tumors. The integrated diagnosis now applies to infiltrating gliomas, a category that includes diffusely infiltrating astrocytoma grades II, III, and IV, and oligodendroglioma, grades II and III, thereby encompassing the most common primary intra-axial central nervous system tumors. Other neoplasms such as medulloblastoma, embryonal tumor with multilayered rosettes, certain supratentorial ependymomas, and atypical teratoid/rhabdoid tumor are also eligible for integrated diagnosis, which can sometimes be aided by characteristic immunohistochemical markers. Since 2016, advances in molecular neuro-oncology have resulted in periodic updates and clarifications to the integrated diagnostic approach. These advances reflect expanding knowledge on the molecular pathology of brain tumors, but raise a challenge in rapidly incorporating new molecular findings into diagnostic practice. This review provides a background on the molecular characteristics of primary brain tumors, emphasizing the molecular basis for classification of infiltrating gliomas, the most common entities that are eligible for an integrated diagnosis. We then discuss entities within the diffuse gliomas that do not receive an integrated diagnosis by WHO 2016 criteria, but have distinctive molecular features that are important to recognize because their clinical behavior can influence clinical management and prognosis. Particular attention is given to the histone H3 G34R/G34V mutant astrocytomas, an entity to consider when faced with an infiltrating glioma in the cerebral hemisphere of children and young adults, and to the group of histologically lower grade diffuse astrocytic gliomas with molecular features of glioblastoma, an important category of tumors to recognize due to their aggressive clinical behavior.

## Background

Primary brain tumors encompass many distinct tumor types arising in the brain parenchyma or meninges, with varying prevalence based on patient age and tumor location. The most common malignant primary brain tumors are gliomas, a category of tumors arising from glial or glial precursor cells that includes astrocytomas (which may be diffuse or circumscribed), oligodendrogliomas, ependymomas, and other rare histologic groups. Together, gliomas account for about 75% of malignant primary brain tumors, and the vast majority are glioblastoma [1]. This review focuses on the molecular features of diffuse gliomas, a category of tumors defined by infiltrating neoplastic cells invading through the brain or spinal cord parenchyma without a distinct margin. Notably, advances in molecular characterization of brain tumors have occurred well beyond this histologic category. Other tumor categories with important molecular pathologic features include the circumscribed astrocytomas such as pilocytic astrocytoma, pleomorphic xanthoastrocytoma, and subependymal giant cell astrocytoma, all of which are more frequent in children and young adults. Embryonal tumors are a category of aggressive, poorly-differentiated tumors, also more common in children, and mostly accounted for by medulloblastomas [2]. Other embryonal tumors such as atypical teratoid/rhabdoid tumor (ATRT) and embryonal tumor with multilayered rosettes (ETMR) are rare, but important to recognize due to their aggressive behavior and distinct molecular underpinnings. Glioneuronal tumors include tumors with a mixture of glial and neuronal differentiation, and as a group they are mostly low grade. Glioneuronal tumors show significant histologic and molecular overlap, which can make the diagnosis challenging [3, 4]. References cited in this article provide details on many of these other entities, and their molecular features.

The article will address the histologic categories of diffuse astrocytoma, anaplastic astrocytoma, glioblastoma, oligodendroglioma, anaplastic oligodendroglioma, and the key histologic and molecular features seen with these morphologies. The diffuse gliomas require molecular information for classification, and these tumors are regularly encountered in routine practice. Understanding the relationship between diffuse glioma histologic and molecular features is critical for recognizing cases that require molecular studies. The morphologic basis for glioma classification was established in 1926 by Bailey and Cushing, who devised a set of histologic categories and introduced a naming convention based on tumor resemblance to normal cellular counterparts in the developing nervous system [5–7]. This work set the foundation for morphologic classification of gliomas, which was the mainstay of classification for almost a century. Brain tumor classification was eventually codified in the 1979 first edition of the World Health Organization (WHO) guidelines for central nervous system (CNS) tumor classification, and subsequent editions were published in 1993, 2000, and the 4th edition in 2007.

**Fig. 1:**
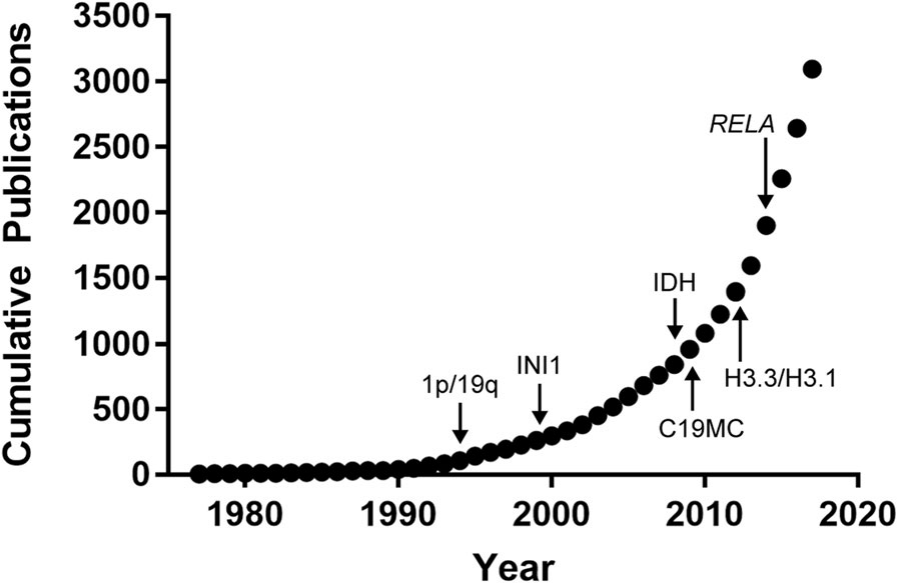
Exponential growth in brain tumor molecular information. Pubmed search results for the combined terms “brain tumor” and “molecular” from 1979 to 2017. Annotations show the year of identification for key molecular features that were incorporated into the WHO 2016 diagnostic recommendations, including 1p/19q-codeletion for oligodendroglioma (1p/19q), loss of INI1 protein for atypical teratoid/rhabdoid tumor (INI1), isocitrate dehydrogenase alterations for lower grade infiltrating gliomas (IDH), histone H3 mutations for diffuse midline and other pediatric high-grade gliomas (H3.3/H3.1), *RELA* fusions for supratentorial ependymoma (RELA), and chromosome 19 microRNA cluster alterations for embryonal tumor with multilayered rosettes (C19MC)

In recent decades, molecular studies of brain tumors resulted in an exponential rise in our knowledge of the molecular underpinnings of these neoplasms (Fig. 1). From the 1998 identification of chromosome arm 1p and 19q loss as a favorable prognostic indicator in infiltrating gliomas and the recognition of the association with oligodendroglioma histology, to the 2008 identification of isocitrate dehydrogenase mutations as an early driver of gliomagenesis, molecular advances have provided critical information that now has implications for brain tumor treatment and prognosis [8–10]. For the infiltrating gliomas, molecular characterization culminated in 2015 with two large studies showing that molecular classification of these tumors more reliably reflected underlying tumor biology than traditional morphology [11, 12]. When lower-grade diffuse gliomas were grouped in an unbiased manner by molecular profiling, key molecular alterations like isocitrate dehydrogenase mutation and chromosome arms 1p and 19q codeletion distinguished the molecular subgroups, but traditional histopathologic features – especially mixed “oligoastrocytoma” morphology – were seen in multiple molecular subgroups [12, 13]. Subsequently, in 2016 a revised 4th edition of the WHO Classification of Tumours of the Central Nervous System (WHO 2016) officially incorporated molecular features into CNS tumor classification [14, 15]. The WHO 2016 added new diagnostic entities, removed others, and updated recommendations on histologic assessment of certain tumors. Many excellent review articles have discussed these revisions, either in general or focusing on gliomas, pediatric tumors, clinical applications, practical approaches to molecular diagnostic testing, and more [14, 16–21].

The aim of this review is to provide an overview of the molecular underpinning of the infiltrating gliomas, the most common brain tumors that should receive an integrated molecular diagnosis by WHO 2016 criteria. Various testing methods exist for obtaining molecular data, and some advantages, limitations, and potential pitfalls of the more common approaches will be discussed. Building on this foundation, we will discuss some molecular entities within infiltrating gliomas that should be considered when a pathologist encounters an unusual tumor that does not seem to fit into an existing molecular category. Molecular pathology of brain tumors as a general topic applies to many other glial and non-glial neoplasms which are not discussed in this review. Readers are referred to several recent focused reviews which have addressed other areas of the molecular pathology of other brain tumors, such as glioneuronal tumors, meningothelial tumors, other mesenchymal tumors, tumors of the sellar region, and lymphomas and histiocytic tumors [20–23].

### The ISN-Haarlem guidelines for reporting an integrated diagnosis

In 2014, the International Society of Neuropathologists (ISN) convened an expert group of over two dozen neuropathologists in Haarlem, the Netherlands, to address challenges in standardization of reporting integrated diagnoses. This group incorporated their expertise with input from over 150 neuro-oncology specialists to set boundaries and priorities for molecular testing in brain tumors. The group prioritized defining entities as narrowly as possible in order to create homogeneous tumor groups, and created a four-layered system to standardize reporting of integrated tumor classification [24]. The ISN-Haarlem system allows separation of the molecular information about a tumor (layer 4), the WHO grade (layer 3), and the histologic classification (layer 2) from the final integrated diagnosis which includes molecular and morphologic characteristics (layer 1), providing more granular information than a single integrated diagnosis alone. This streamlines reporting for cases where the histologic classification appears to conflict with the tumor grade, such as in diffuse midline glioma with histone H3 K27M-mutation, which is considered WHO grade IV based on molecular findings, even with lower-grade histology.

Molecular testing will not be possible in all cases, as tissue quality and quantity are frequent limiting factors in brain tumor sampling and testing may subject to technical failure. The WHO does not endorse specific testing modalities, and each institution can choose from several platforms or approaches [15]. Still, some centers may not have access to the necessary testing to support an integrated diagnosis. The WHO classification still allows reporting of a histologic diagnosis followed by the qualifier “not otherwise specified” (NOS) to reflect that complete molecular information is not available, or testing cannot be performed [15, 25]. In cases where molecular information is obtained, but the results do not fit into an existing diagnostic category, a different qualifier of “not elsewhere classified” (NEC) may be applied [25]. Such cases are presumed to be provisional, and this category should be used with decreasing frequency over time, with expanding molecular studies of brain tumors.

### cIMPACT-NOW: a vehicle for updates to CNS tumor molecular diagnostics

The Consortium to Inform Molecular and Practical Approaches to CNS Tumor Taxonomy (cIMPACT-NOW) was established to create a mechanism for updates in between WHO editions, which have historically been separated by intervals of at least 7 years [26, 27]. The consortium created working committees to address specific questions about molecular diagnosis. The consortium has released guidelines on topics such as how to use the NOS and NEC qualifiers in diagnostic reports, requirements for 1p/19q testing in infiltrating gliomas with astrocytic morphology, and the criteria for the diagnosis of diffuse midline glioma, H3 K27M-mutant, WHO grade IV [25, 28]. A critical and very recent update concerns the molecular criteria that indicate aggressive behavior in IDH-wildtype diffuse or anaplastic astrocytoma, which is discussed later in this review [29]. Updates from cIMPACT-NOW are to be reported in the journal *Acta Neuropathologica* with an accompanying editorial in *Brain Pathology*, and both will be a valuable resource for keeping up to date on molecular pathology of brain tumors.

### Histologic classification and grading of diffuse gliomas

**Fig. 2:**
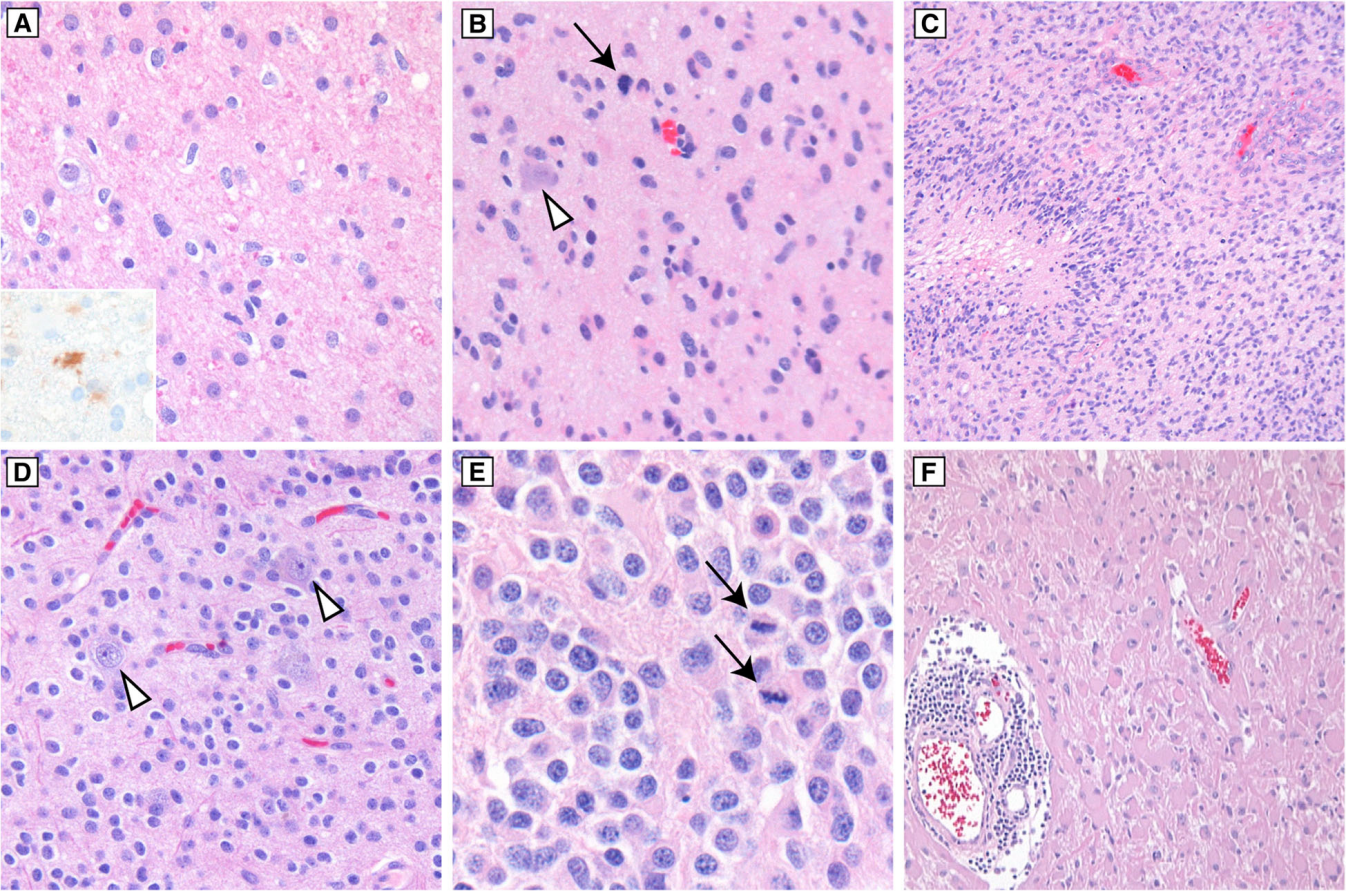
Typical histologic features of infiltrating gliomas. In diffuse astrocytomas (**a**), cellularity is increased due to infiltrating neoplastic astrocytes with irregular, hyperchromatic nuclei and scant associated cytoplasm. Immunohistochemistry for IDH1 R132H mutant protein (A, inset) can be helpful when infiltrating cells are sparse or rare. **b** Anaplastic astrocytoma is distinguished by mitotic activity (black arrow). Note the infiltrating tumor cells around a non-neoplastic neuron (white arrowhead). **c** Palisading necrosis (left) and endothelial proliferation (upper right) are histologic features of glioblastoma, though neither feature is absolutely specific. **d** WHO grade II oligodendroglioma with uniform, rounded nuclei and perinuclear clearing, the latter feature being an artifact of formalin fixation. Infiltrative growth is demonstrated by entrapped non-neoplastic neurons (white arrowheads). **e** Oligodendrogliomas with high cellularity, cytologic atypia, significant mitotic activity (black arrows) generally defined as 6 or more mitoses per 10 high-power fields, and vascular proliferation qualify for anaplastic oligodendroglioma, WHO grade III. Necrosis often accompanies vascular proliferation and mitotic activity, but is not required. **f** Gemistocytic astrocytoma appears as cells with abundant, glassy, eosinophilic cytoplasm, and frequently is associated with perivascular inflammatory infiltrates. This astrocytoma subtype has a propensity toward rapid malignant progression

Diffusely infiltrating gliomas are defined by a growth pattern of individual tumor cells growing through the brain parenchyma, in contrast with the sharp, pushing border seen in circumscribed gliomas or metastatic tumors [30]. Individual tumor cells may be seen surrounding entrapped neurons, clustering around small vessels, and accumulating in the subpial space. These “secondary structures” are highly specific for diffuse glioma. Conventional astrocytic histologic features are elongated, hyperchromatic tumor cell nuclei with irregular nuclear contours and scant associated cytoplasm (Fig. 2, a-c). Oligodendroglial features include uniform, round to oval nuclei with crisp nuclear borders, delicate speckled chromatin, and (in formalin fixed tissue) perinuclear cytoplasmic clearing, with a background of delicate branching small vessels (Fig. 2, d-e) [31]. Diffuse glioma grading is based on mitotic activity, vascular proliferation, and necrosis. Diffuse astrocytomas with significant mitotic activity are considered WHO grade III (anaplastic) while glioblastoma, WHO grade IV, is defined by the presence of vascular proliferation and/or necrosis, the latter feature being often (but not always) palisading (Fig. 2, b-c). Conventional oligodendroglioma is WHO grade II, while findings of high cellularity, cytologic atypia, necrosis, vascular proliferation, and significant mitotic activity are features for designating anaplastic oligodendroglioma, WHO grade III, with a minimum of “conspicuous microvascular proliferation and/or brisk mitotic activity” required for the diagnosis (Fig. 2, d-e) [15, 32]. Gemistocytic astrocytoma is a variant characterized morphologically by at least 20% of the neoplastic cells showing abundant eosinophilic cytoplasm and short branching processes (Fig. 2f). These tumors may have a more aggressive course and frequently show copy number gain of chromosome arm 12p encompassing the cyclin D2 (*CCND2*) locus [33]. A few glioblastoma histologic subtypes are recognized as distinct entities by the 2016 WHO, such as epithelioid glioblastoma, giant cell glioblastoma, and gliosarcoma. Epithelioid glioblastoma is a new addition to the WHO 2016 [15]. These tumors mostly occur in younger patients, and about half show *BRAF* V600E mutations; the differential diagnosis with anaplastic pleomorphic xanthoastrocytoma can be challenging and the relationship between these entities is controversial [34, 35]. Other histologic subtypes are considered morphologic variants, such as small cell glioblastoma, and glioblastoma with primitive neuronal component. These entities and their characteristic histologic features have been reviewed elsewhere [31].

While some infiltrating gliomas readily declare themselves morphologically as astrocytoma or oligodendroglioma, many are ambiguous and appear either as a mixture of both cell types, or with a constellation of nuclear features that defies reproducible categorization. Before 2016, such “in-between” diffuse gliomas were categorized as “oligoastrocytoma” and assigned WHO grade II, or grade III (anaplastic) in the setting of significant mitotic activity, necrosis, and/or vascular proliferation. Oligoastrocytomas had an intermediate prognosis between histologically defined diffuse astrocytomas and oligodendrogliomas, but diagnostic reproducibility for this category or tumors was poor [36, 37]. The WHO 2016 eliminated oligoastrocytoma as a distinct entity, since nearly all histologically defined oligoastrocytomas can be re-categorized as oligodendroglioma or astrocytoma based on molecular features [38]. Similarly, nearly all anaplastic oligoastrocytomas and glioblastomas with oligodendroglial component (essentially, a WHO grade IV oligoastrocytoma) declare themselves by molecular criteria as either anaplastic oligodendroglioma, anaplastic astrocytoma, or glioblastoma [39]. There are reports of diffuse gliomas with distinct oligodendroglial and astrocytic components by morphologic and molecular criteria, but such cases are exceptionally rare, and these are not recognized as a distinct entity by current criteria [40, 41]. The WHO recognizes that in some diffuse glioma cases where molecular testing cannot be done, a tumor may fall into the old oligoastrocytoma category. In such cases, the diagnosis of “oligoastrocytoma, NOS” can be made, though such cases should be rare. If molecular testing results do not fit with an existing molecular category, the diagnosis of “oligoastrocytoma, not elsewhere classified” may be applied [25].

### Molecular classification of lower grade infiltrating gliomas: IDH mutations and 1p/19q-codeletion

In 2008, next-generation sequencing identified recurrent mutations in isocitrate dehydrogenase 1 (encoded by *IDH1*) in a subset of glioblastoma cases, mostly from younger patients with a history of prior lower-grade astrocytoma (clinically referred to as secondary glioblastoma) [9]. Subsequent studies showed that recurrent missense mutations involve *IDH1* at position arginine 132, and less commonly in the homologous arginine 172 of *IDH2*. Overall, *IDH1* R132 and *IDH2* R172 alterations occur in about 80–90% of adult WHO grade II or III infiltrating astrocytomas, oligodendrogliomas, and secondary glioblastoma [42]. Normally, the isocitrate dehydrogenase proteins -- collectively referred to as IDH -- catalyze the oxidative decarboxylation of isocitrate to α-ketoglutarate. The mutant forms of IDH acquire a neomorphic activity and instead convert isocitrate to D-2-hydroxyglutarate, a so-called “oncometabolite” that builds to a very high level in tumor cells and interferes with several cellular processes, ultimately resulting in changes to DNA and histone methylation patterns that alter gene expression by establishing a glioma CpG island methylator phenotype (G-CIMP) [43, 44]. The most common form of mutant IDH is a missense mutation in *IDH1* converting arginine at position 132 to histidine (IDH1 R132H). This mutant protein is detectable by a sensitive and specific antibody [45, 46]. Immunopositivity for IDH1 R132H is sufficient to classify a glioma as “IDH-mutant” [15]. If the immunostain is negative, sequencing of *IDH1* and *IDH2* can be performed to assess for less common *IDH1* mutations, or mutations in *IDH2*. These non-canonical alterations are very rare in patients over 54, so sequencing is generally not necessary in older patients with glioblastoma histology and no history of a prior lower grade glioma [25, 47, 48]. If sequencing cannot be performed or the necessary assays have failed, tumors receive a histologic diagnosis and the NOS qualifier.

**Fig. 3:**
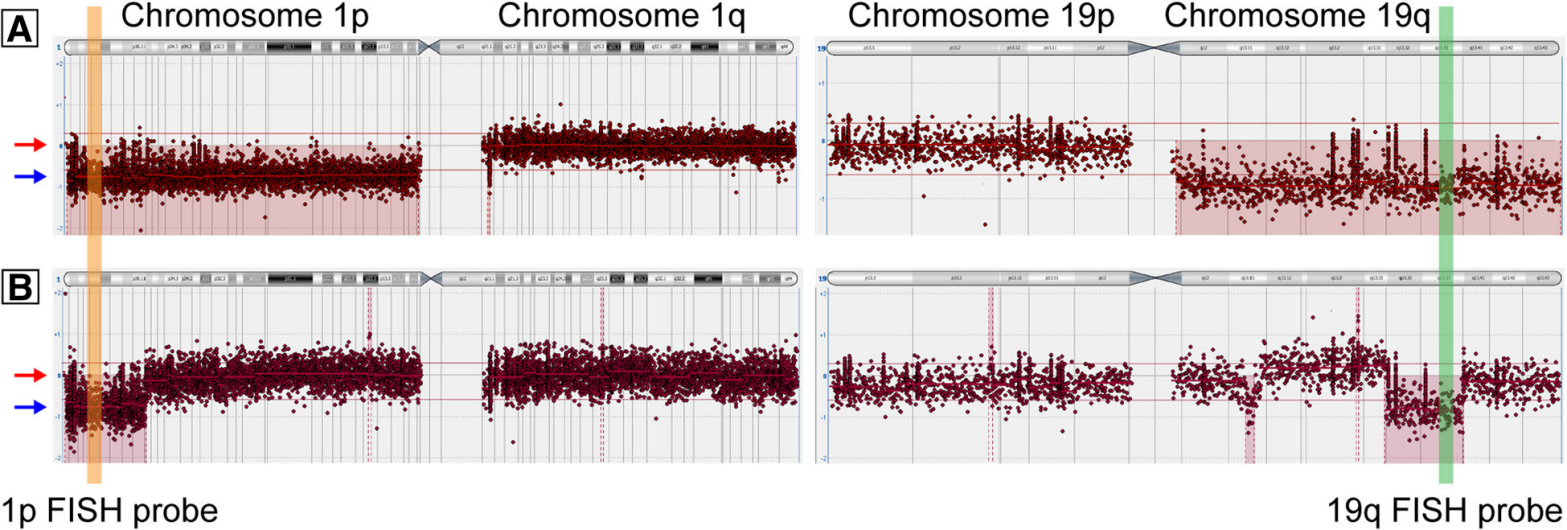
Assessment for whole-arm 1p and 19q loss. Examples of single nucleotide polymorphism chromosomal arrays (SNP microarrays) for (**a**) IDH-mutant oligodendroglioma and (**b**) IDH-wildtype glioblastoma. Red arrows mark a neutral copy number, and blue arrows accompanied by red shading indicate areas of copy number loss. Note that in oligodendroglioma, the entire 1p and 19q arms show copy number loss. In contrast, for the glioblastoma, there is only focal loss of distal 1p, and a focal interstitial loss on 19q, with these areas encompassing the region that corresponds to FISH probes for 1p (orange shading) and 19q (green shading). The probe annealing regions are not to scale

Oligodendrogliomas now have a strict molecular definition and must show an IDH alteration and evidence for deletion of both the short arm of chromosome 1 (1p) and the long arm of chromosome 19 (19q) for an integrated diagnosis. 1p/19q-codeletion results from a reciprocal translocation between chromosomes 1 and 19 -- t(1;19)(p10;q10) -- with subsequent loss of one derivative chromosome, leaving an imbalance and loss of 1p and 19q [49, 50]. For many years, fluorescence in situ hybridization (FISH) has been used to define the 1p/19q-codeletion, using molecular probes that align at the distal ends of the chromosome 1 and 19 arms. For example, probes from Abbott Molecular are located at 1p36.2/1q25.2 and 19p13.2/19q13.3, and the ratio of 1p to 1q and 19q to 19p is calculated to determine deletion. Recently, evidence has amassed that FISH is insufficient to fully distinguish oligodendrogliomas from other brain tumors (usually glioblastoma) that harbor focal deletions of 1p and 19q and thus give false positive results on FISH analysis [51, 52]. For this reason, the WHO 2016 recommends molecular testing by a method that assess whole-arm chromosomal loss, such as molecular inversion probe array, single nucleotide polymorphism chromosomal microarrays (hereafter abbreviated SNP microarrays), or next-generation sequencing with copy number analysis [20]. Our molecular diagnostics group has come to a similar conclusion, and whenever possible our glioma testing is now performed by SNP microarrays, occasionally showing that results of “1p/19q-codeletion” by FISH are due to focal terminal or interstitial deletions rather than whole arm losses (Fig. 3, a-b). Another advantage is that this platform assesses genome-wide copy number changes, and alterations such as *EGFR* amplification, focal deletions on 9p encompassing *CDKN2A/B*, and focal or whole chromosome gains/losses can be detected. For laboratories not currently offering microarray methods, it may be possible to include additional, more proximal FISH probes (i.e. closer to the centromere) to increase confidence in whole arm codeletion, although this approach would increase technical and scoring complexity and potentially increases cost to a similar level as microarray platforms.

### The role of telomere maintenance: ATRX, p53, and the *TERT* promoter

Replicative senescence occurs as a consequence of cell division. Incomplete replication of the chromosome ends leads to progressive shortening of the telomeres, eventually triggering a DNA damage checkpoint signal resulting in cell cycle arrest [53]. Telomere length is maintained in stem/progenitor and germline cells by telomerase [53]. The telomerase holoenzyme consists of telomerase reverse transcriptase or TERT, a catalytic subunit encoded by the *TERT* gene on chromosome 5, dyskeratin, and template telomerase RNA component, which together act to extend telomeres through addition of a repetitive DNA sequence to the chromosome ends [53, 54]. TERT expression is normally silenced in nearly all somatic cells, and a telomerase reactivation is an important factor in escape from replicative senescence, one of the hallmarks of cancer [55]. Recurrent, mutually exclusive point mutations in the *TERT* promoter were originally discovered in melanoma, and subsequently identified in other tumors, including at a high frequency in primary glioblastoma and oligodendroglioma [56–58]. Mechanistic studies later showed that *TERT* promoter mutations generate a cryptic binding site for an E26 transformation-specific (ETS) family transcription factor called GA-binding protein, alpha subunit (GABPA), leading to telomerase re-expression and subsequent telomere elongation [59]. Sequencing of the *TERT* promoter region is the only method to detect this alteration; in several studies of multiple cancer types, including gliomas, TERT immunohistochemistry does not correlate with promoter mutation status [56, 60–62]. Epigenetic mechanisms can also regulate *TERT* expression, and this may have prognosis in certain pediatric brain tumors, where *TERT* promoter mutations are rare [57, 63, 64]. Alternative mechanisms for telomere maintenance include *TERT* amplification and *TERT* promoter rearrangements [65, 66].

In contrast to oligodendrogliomas and IDH-wildtype glioblastoma, *TERT* promoter mutations are rare in IDH-mutant astrocytomas, which instead maintain telomere length through a mechanism involving mutations in the *ATRX* gene, usually accompanied by mutations in *TP53*. *ATRX* encodes α-thalassemia/mental retardation syndrome X-linked, a protein that, along with death-domain associated protein or DAXX, is involved in maintaining chromatin structure at the telomeres [54]. *ATRX* alterations strongly correlate with a phenotype called alternative lengthening of telomeres (ALT), characterized by increased telomere homologous recombination and subsequent telomere elongation [67]. Within brain tumors, the ALT phenotype is frequent in *IDH*-mutant astrocytomas and histone H3 mutant gliomas.

**Fig. 4:**
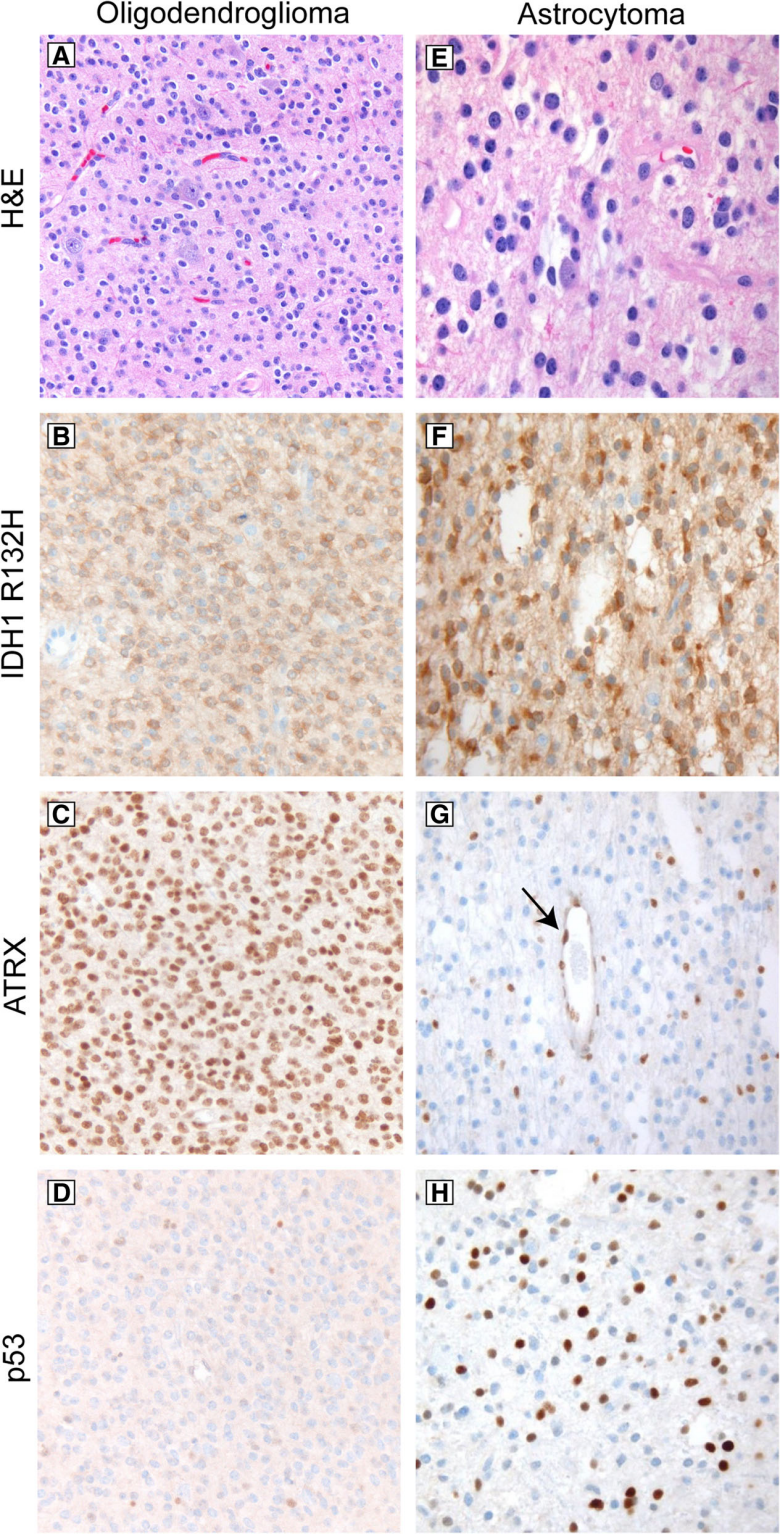
Immunohistochemical features in typical lower grade infiltrating gliomas. Oligodendrogliomas (**a**) are immunoreactive for IDH1 R132H (**b**) unless they have a less common non-canonical mutation, in which case sequencing of *IDH1* and *IDH2* is required; they lack *ATRX* mutations and therefore retain nuclear reactivity of ATRX protein in tumor cell nuclei (**c**), and p53 immunoreactivity is generally low (**d**), except in anaplastic cases. Astrocytomas with IDH mutation (**e-f**) and *ATRX* mutations almost always show loss of ATRX staining in tumor nuclei, while endothelial cells (black arrow) and entrapped normal cells are positive, providing an internal positive control (**g**). *TP53* mutations are associated with increased staining in tumor cell nuclei (**h**)

Immunohistochemical markers provide insight into underlying molecular alterations and aid in glioma classification (Fig. 4) [17, 68]. Typical oligodendrogliomas are positive for IDH1 R132H unless they have a non-canonical IDH alteration, and generally show strong nuclear staining for ATRX and little to no nuclear reactivity for p53 (Fig. 4, a-d), except for anaplastic cases which can show a significant amount of p53 immunoreactivity. In astrocytomas, *ATRX* mutations are usually accompanied by loss of nuclear staining for ATRX protein, and *TP53* mutations result in stabilization of the p53 protein leading to strong nuclear staining in a significant proportion of tumor nuclei (Fig. 4, e-h). ATRX immunoreactivity can be patchy because the stain is very sensitive to fixation and cautery, so before concluding that a diffuse glioma shows ATRX loss, internal positive control staining in the endothelial, inflammatory, and entrapped neuronal component in the same part of the sample should be identified (Fig. 4, g). Also, not all *ATRX* mutations are associated with loss of nuclear staining, so in a tumor with classic astrocytoma histology and a confirmed *IDH1* or *IDH2* mutation, positive nuclear staining for ATRX does not exclude the diagnosis of astrocytoma.

Immunohistochemistry for p53 bears some caveats. Most *TP53* mutations are missense mutations that result in nuclear accumulation of the protein [69]. However, this immunostaining pattern is neither sensitive nor specific for a *TP53* mutation and the result must be interpreted in context with morphology and other immunohistochemical and molecular findings. In one series of 157 diffuse gliomas, a cutoff of strong nuclear p53 reactivity in > 10% of tumor nuclei had a positive predictive value of 94.5% and negative predictive value of 86.3% for predicting *TP53* mutation status [69]. Within brain tumors, *TP53* mutations can be seen in *IDH*-wildtype glioblastomas, occasional circumscribed gliomas, embryonal tumors including medulloblastoma, other rare primary brain tumors such as choroid plexus neoplasms, and in metastatic tumors. Also, non-neoplastic conditions -- especially demyelinating processes -- can be associated with increased p53 nuclear staining, and this can be a particular pitfall in progressive multifocal leukoencephalopathy since that condition can cause significant astrocytic atypia [70]. Finally, absence of nuclear p53 staining does not exclude a *TP53* mutation since truncating or splice site *TP53* mutations are a mechanism for loss of p53 function, and this leads to loss of nuclear immunoreactivity due to decreased or absent protein expression [69]. Overall, with the appropriate tumor morphology of a diffuse or anaplastic astrocytoma, either ATRX loss or strong p53 staining in > 10% of tumor nuclei is sufficient to diagnose an astrocytoma of the appropriate grade without assessing 1p/19q [28]. A combination of tumor morphology, IDH status, and the immunophenotype for IDH1-R132H and ATRX, aided by p53, is adequate to classify most diffusely infiltrating gliomas, with confirmatory assessment of 1p/19q status in suspected oligodendrogliomas and sequencing of the IDH genes to detect suspected non-canonical alterations, and these modalities can be applied in a sequential or algorithmic approach [71, 72].

### Molecular characteristics of IDH-wildtype glioblastoma

Most glioblastomas occur in adults, and 90% are IDH-wildtype tumors that arise de novo, i.e. with a rapid clinical presentation and absent a precursor lower-grade lesion (primary glioblastoma). The remainder (about 10%) are mostly IDH-mutant tumors, typically arising from lower grade infiltrating astrocytomas, and thus clinically referred to as secondary glioblastoma. The molecular features of IDH-wildtype glioblastoma have been studied extensively, however the prognosis remains poor [1]. Amplification of double minute chromosomes of the epidermal growth factor receptor gene (*EGFR*) is a frequent event in IDH-wildtype glioblastomas, while *EGFR* point mutations are comparatively rare. The *EGFR* gene also commonly incurs an intragenic deletion of exons 2–7 producing a constitutively active variant protein called EGFR-vIII, occurring in a quarter to half of glioblastoma. Expression of EGFR-vIII is almost always associated with amplification of the wild-type *EGFR* allele, but the clinical significance of either alteration is still unclear [73, 74]. Concomitant chromosome 7p gain combined with chromosome 10q loss is the most frequent genetic alteration in glioblastoma, with almost half showing both alterations; a large fraction of *EGFR* amplification occurs on a background of chromosome 7 polysomy and chromosome 10 monosomy [75, 76].

The O^6^-methylguanine-DNA methyltransferase (MGMT) protein, encoded by the *MGMT* gene on chromosome 10, plays a role in repairing the DNA damage from alkylating agents, including temozolomide. Methylation of the *MGMT* promoter region is associated with silencing of gene expression, and occurs in about 40–50% of glioblastoma cases [15]. Mechanistic studies suggest that tumors with low MGMT are deficient in repairing temozolomide-induced DNA damage and therefore have higher chemosensitivity. In 2005, results from a randomized multicenter phase III clinical trial comparing radiotherapy to radiotherapy with concomitant temozolomide demonstrated *MGMT* promoter methylation as an independent favorable prognostic factor, and a predictive factor for survival benefit in patients treated with temozolomide and radiation therapy [77, 78]. Subsequent studies support *MGMT* promoter methylation as a predictive biomarker in glioblastoma, but the exact role in guiding clinical management is still being refined, partly due to challenges in testing methodology and establishing meaningful cutoffs for the assays [79].

### Molecular methods in IDH-wildtype glioblastoma

FISH readily identifies *EGFR* amplification, but is being phased out in favor of chromosomal microarray, which can detect the amplification with better total copy number estimates and provides additional copy number information across the genome. The deleted region for EGFR-vIII is small, at only 13 kilobases, and therefore not amenable to FISH, and furthermore mosaicism complicates microarray detection and interpretation, particularly against the background of an amplified gene. Currently, of the genomic technologies readily available in the lab, next generation sequencing with high read depth is the most robust approach for detecting this deletion. Partial as well as whole chromosome gains and losses are readily observed using SNP microarray, which can circumvent some of the limitations of targeted FISH. For example, homozygous deletion of the *DMBT1* gene on chromosome 10 (encoding Deleted In Malignant Brain Tumors 1 protein) has been reported in glioblastoma and medulloblastoma [80]. In tumors with diffuse astrocytoma histology, *DMBT1* loss may be predictive of a poor outcome, although IDH status was not assessed in that study so the status of *DMBT1* as an independent predictor of outcome is unclear [81]. *DMBT1* is located at chromosome 10q26.31, approximately 35 megabases distal to *PTEN*. Traditional FISH using a *PTEN* probe will not detect *DMBT1* deletion; SNP microarray will.

Copy number neutral loss of heterozygosity (LOH) is another common event in glioblastoma and can be detected using SNP chromosomal microarray [82]. LOH can uncover recessive mutations due to the transfer of one recessive allele onto both chromosome homologues via chromosomal crossover events, and LOH of 17p, including the *TP53* gene, is a frequent event [22]. Regional LOH may also prove useful in “gene discovery” efforts by identifying regions where a tumor suppressor or oncogene may reside. Additionally, SNP patterns may be more informative than oligonucleotides for large copy number changes only impacting a minority of cells, as the SNPs tend to be more sensitive to subtle changes associated with mosaicism. Finally, the use of both oligonucleotides and SNP microarrays has the advantage of one modality confirming the other, since the hybridizations are independent. Without micro-dissection in samples with a small tumor fraction (e.g. < 20%), it becomes difficult to detect small changes (e.g. < 20 Mb) on microarray and FISH is a more sensitive technique. Thus, expert pathology review is critical for microarray studies, just as it has historically been for FISH.

Molecular methods for *MGMT* promoter methylation testing include simple or quantitative methylation-sensitive polymerase chain reaction, pyrosequencing, multiplex ligation-dependent probe amplification, and analysis by DNA methylation array. The relative costs and advantages/disadvantages of each platform were recently reviewed [79]. Our institution uses pyrosequencing, but other methods could be chosen depending on specimen volume, testing costs, and other laboratory/technical factors.

### Histone H3 mutant gliomas

Histones are a family of proteins that form a protein/DNA complex to maintain chromatin structure and regulate gene expression, which can be mediated by post-translational histone modifications including methylation and acetylation. Histone octamers comprise two each of H2A, H2B, H3, and H4 proteins, and these are encoded by different genes. The histone H3.3 variant is expressed throughout the cell cycle and is encoded by *H3F3A* and *H3F3B*, while the histone H3.1 variants are highly expressed during DNA replication and is encoded by several genes including *HIST1H3B* and *HIST1H3C*. Two categories of histone mutations have been identified in brain tumors. Sequencing studies of pediatric diffuse intrinsic pontine gliomas and non-brainstem glioblastomas identified recurrent mutations causing a lysine to methionine substitution at position 27 -- hereafter K27M -- in *H3F3A* and *HIST1H3B/C*, which are hereafter grouped as H3 to encompass H3.3 and H3.1 [83, 84]. Very rare alternative alterations include a lysine to isoleucine alteration in *H3F3A*, and a K27M alteration in histone H2 variant encoded by *HIST2H3C* [85]. The H3 K27M mutant protein has a dominant negative effect on the enhancer of zest 2 (EZH2) methyltransferase protein, which is a component of the polycomb repressive complex normally responsible for H3 K27 trimethylation [86]. Since the methionine residue of H3 K27M cannot be methylated, and also inhibits EZH2, the result is global alterations histone methylation and consequent dysregulation of gene expression [86]. The H3 K27M alteration has since been identified in midline gliomas across a spectrum of ages and tumor locations, including the third ventricle, pineal region, cerebellum, and spinal cord [87]. The WHO 2016 recognizes H3 K27M-mutant diffuse gliomas as a distinct molecular entity, and importantly these tumors correspond to WHO grade IV regardless of their histologic grade. The integrated diagnosis for these tumors is “Diffuse midline glioma, H3 K27M-mutant, WHO grade IV”. Using the ISN-Haarlem system, this integrated diagnosis can go along with a histologic classification of diffuse astrocytoma, anaplastic astrocytoma, or glioblastoma, illustrating the usefulness of the layered reporting system since under different molecular circumstances a diffuse or anaplastic astrocytoma is WHO grade II or III, respectively.

**Fig. 5:**
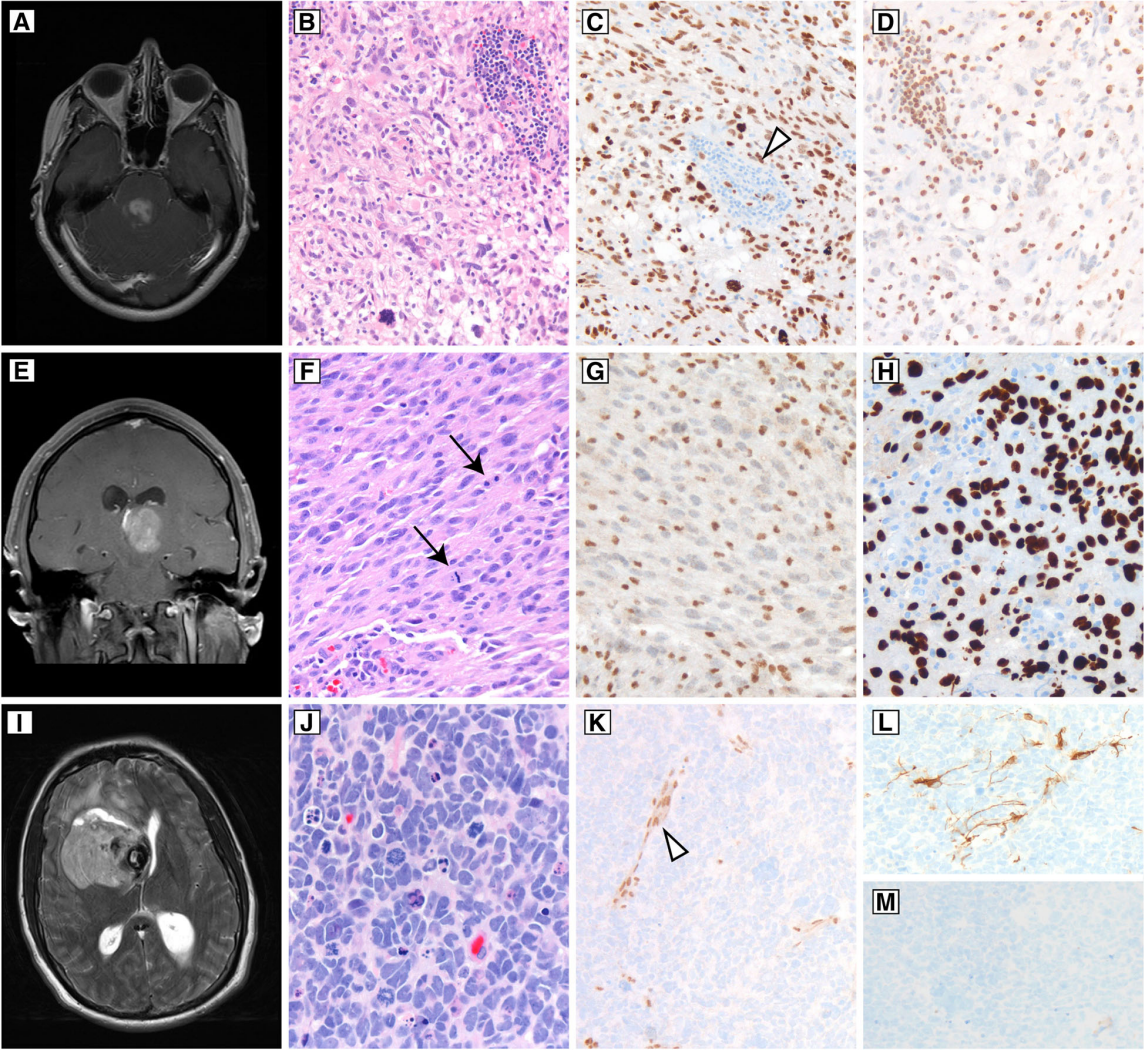
Examples of histone H3 mutant gliomas. **a** Post-contrast T1-weighted imaging of an enhancing pontine mass occurring in a 33-year-old woman. Biopsy showed a markedly pleomorphic astrocytoma with frequent mitotic figures and prominent perivascular inflammation (**b**). Tumor cell nuclei were positive for histone H3 K27M mutant protein (**c**), while inflammatory cells were negative for this marker (white arrowhead). Immunohistochemistry for trimethylated histone H3 is negative in tumor nuclei (**d**), and appropriately stains inflammatory cells. **e** Post-contrast T1-weighted imaging of a left thalamic tumor occurring in a 31-year-old man who presented with headaches. Sections showed an astrocytic neoplasm with mitotic activity (black arrows) (**f**). ATRX was absent in tumor cell nuclei, and stained a background population of macrophages and microglial cells (**g**). An immunostain for H3 K27M was positive in tumor nuclei (**h**), supporting the diagnosis of diffuse midline glioma, H3 K27M-mutant, WHO grade IV. (**i**) T2-weighted imaging of a right frontal cerebral hemispheric tumor in a 35-year-old man who presented with headaches. Sections showed a tumor with brisk mitotic activity, frequent apoptotic cells, high nuclear to cytoplasmic ratios, and nuclear molding (**j**). ATRX was negative in tumor cell nuclei (**k**), with preserved staining in the endothelial cells (white arrowhead). GFAP showed only focal staining (**l**), and OLIG2 was completely negative (**m**). p53 (not pictured) was strongly positive. Sequencing of this tumor showed wildtype *IDH1* and *IDH2*, and a further sequencing study revealed an *H3F3A* G34R mutation

Three examples of histone H3 mutant glioma are presented in Fig. 5, with H3 K27M-mutant tumors of the midbrain and thalamus presented in panels A-D and E-H, respectively. The H3 K27M mutant protein is detectable by a highly sensitive and mutation-specific antibody (Fig. 5, c and h) [88]. However, since the K27M epitope is in a highly conserved region of the histone H3 family, the antibody does not distinguish mutations in *H3F3A* (H3.3-mutant) from *HIST1H3B/C* (H3.1-mutant). This distinction may impact prognosis and eligibility for clinical trials, and in such cases sequencing of *H3F3A, HIST1H3B/C*, and possibly *HIST2H3C* may be required [85, 88, 89]. Because the H3 K27M mutant protein suppresses histone H3 trimethylation by mechanisms described above, expression of H3 K27M is associated with global reduction of histone H3 lysine position 27 trimethylation (H3 K27me3). This change is also detectable by immunohistochemistry (Fig. 5d), which reveals global loss of nuclear H3 K27me3 reactivity in tumor cells, however this is less specific than expression of the mutant protein, with the most common pitfall being focal loss of H3 K27me3 in atypical teratoid/rhabdoid tumor [88]. Importantly, global loss of histone H3 K27me3 is also an emerging prognostic indicator for an aggressive subgroup of posterior fossa ependymomas [90, 91]. The underlying mechanism for reduced H3 K27me3 in some ependymomas is unclear, but it occurs without the H3 K27M mutations that define diffuse midline gliomas. In practice, most H3 K27M-mutant diffuse midline gliomas will show both positive nuclear staining for H3 K27M mutant protein, and a corresponding loss of nuclear staining for H3 K27me3. For the mutation-specific antibody, the stain should show strong nuclear staining in tumor nuclei, and normal cellular components such as endothelial cells, inflammatory cells, and entrapped neurons should be negative (Fig. 5, c and h). Granular cytoplasmic staining is considered nonspecific. For H3 K27me3, non-neoplastic cellular components should be nuclear positive, and the tumor nuclei should be negative (Fig. 5d).

When the WHO 2016 was published, H3 K27M mutations were thought to be specific for diffuse midline gliomas. However, H3 K27M mutations have now been reported in midline circumscribed gliomas such as ganglioglioma, pilocytic astrocytoma, unspecified glioneuronal tumors, and ependymoma, and in some series the clinical behavior of these tumors is more aggressive than histologically comparable H3 K27-wildtype counterparts, although it is not as dismal as for diffuse gliomas [92–95]. Intriguingly, H3 K27M mutations can co-occur with *BRAF* V600E, a common driver alteration in a variety of circumscribed gliomas and glioneuronal tumors [19, 94]. In practice, this means that evidence for a H3 K27M mutation does not necessarily define the integrated diagnosis of diffuse midline glioma, WHO grade IV. The histologic context still matters: tumors must be definitively *midline, diffusely infiltrating gliomas with a K27M alteration* to qualify for the integrated diagnosis [28]. Due to the data supporting a worse clinical outcome of these rare H3 K27M-mutant circumscribed gliomas, our practice is to evaluate any midline ganglioglioma or pilocytic astrocytoma for H3 K27M mutant protein expression since these are the most common circumscribed histologic entities where H3 K27M has been reported.

*H3F3A* mutations also occur at guanine position 34, substituting arginine (G34R) or valine (G34V – hereafter G34R/V) in histone H3.3, and an example of this is provided in Fig. 5, panels I through M. Unlike H3 K27M, the H3.3 G34R/V alteration has not been identified in the histone H3.1 isoforms encoded by *HIST1H3B/C*. The H3.3 G34R/V mutant tumors typically occur in the cerebral hemispheres in adolescents and young adults, and in a series of 81 cases almost always show loss of ATRX by immunohistochemistry (95%), as well as *TP53* mutation (88%) [96]. Since the H3.3 G34R/V alteration is mutually exclusive with IDH mutations, H3.3 G34R/V should be considered in a hemispheric diffuse glioma that shows loss of ATRX but is proven by sequencing to be *IDH1* and *IDH2* wildtype. About one third of the H3.3 G34R/V mutant tumors show primitive neuronal features, with monomorphic cells showing a high nuclear to cytoplasmic ratio and largely lacking vascular proliferation or necrosis, and regardless of their histologic pattern (i.e. primitive neuronal or astrocytic) GFAP and OLIG2 expression may be limited (Fig. 5, j, l, and m) [96, 97]. An H3.3 G34R/V mutant tumor should also be considered in the differential diagnosis of a central nervous system primitive neuronal tumor, even if the typical glial markers are negative or focal/patchy. Most H3.3 G34R/V mutant tumors are *MGMT* promoter hypermethylated, which may contribute to a slightly better outcome in this group of tumors [96]. Since the H3.3 G34R/V is not currently a separate integrated diagnostic entity, these cases would be reported as astrocytomas of the appropriate histologic grade along with the “not elsewhere classified” modifier, and the molecular result of *H3F3A* G34R/V status should be reported [25]. The more general designation of astrocytic glioma, IDH-wildtype is not applicable since proven H3 G34R/V-mutant diffuse gliomas have another disease-defining molecular alteration and distinct clinical features [29]. Mutation-specific antibodies to detect H3.3 G34R or G34V mutant protein are in use at a few academic centers.

### Diffuse astrocytic glioma with molecular features of glioblastoma, WHO grade IV

The WHO 2016 classifies glioblastoma into IDH-mutant and IDH-wildtype categories. This distinction has clinical utility in that multiple studies showed significantly better outcomes in IDH-mutant glioblastoma [13, 98]. In either molecular category, a tumor must still meet the appropriate histologic criteria to be diagnosed as a morphologic glioblastoma - namely tumors must show predominantly astrocytic differentiation with an infiltrative growth pattern, and exhibit microvascular proliferation and/or necrosis. Histologic parameters do not reliably distinguish IDH-wildtype from IDH-mutant tumors, although one recent and intriguing study showed that microthrombi are predictive of IDH-wildtype status [31, 99]. Prior to and since the publication of the WHO 2016, studies have shown that some infiltrating astrocytomas that do not meet histologic criteria for glioblastoma may clinically behave in a manner similar to histologically defined glioblastoma. In particular, IDH-wildtype infiltrating astrocytomas, which would be histologically classified as grade WHO grade II or III, in many cases have worse outcomes than IDH-mutant glioblastomas, which are histologically grade IV, creating a significant problem in the current grading criteria [98, 100, 101].

The c-IMPACT-NOW consortium recently addressed this diagnostic dilemma by recommending a set of molecular features that indicate aggressive behavior in histologically lower grade, IDH-wildtype diffuse astrocytic gliomas [29]. Per these recommendations, diffuse astrocytic gliomas qualify for this designation with evidence of one or more of the following molecular alterations: (1) *EGFR* amplification, (2) *TERT* promoter mutation, and (3) whole-chromosome 7 gain combined with whole chromosome 10 loss (hereafter + 7/− 10). Patients with a proven IDH-wildtype, histologically grade II or III infiltrating astrocytoma and any of these alterations can be diagnosed with “Diffuse astrocytic glioma, IDH-wildtype, with molecular features of glioblastoma, WHO grade IV”, reflecting that their tumors are likely to behave in a manner similar to IDH-wildtype glioblastoma. These alterations may be seen alone or in combination, and they have varying sensitivity and specificity as individual parameters or in combination. Stitchel et al recently described these alterations across a large cohort of brain tumors that were categorized by global DNA methylation profiling (discussed below) [102]. DNA methylation profiling is an exciting technology with great promise for brain tumor classification and identifying molecular subgroups within and between histopathologic entities, and the tools for this analysis are publicly accessible through the German Cancer Research Center web portal at www.molecularneuropathology.org [103, 104]. As a molecular signature of glioblastoma in that series, *EGFR* amplification is the most specific but least sensitive marker, with 99.8% specificity and 36.0% sensitivity. In this context, *EGFR* amplification refers to high level copy number gains; low level copy number gains and immunohistochemical expression of EGFR are not sufficiently specific for this purpose [105]. The + 7/− 10 chromosomal alteration is also relatively specific (98.0%) and was more sensitive that *EGFR* amplification (59.4%). Although partial arm deletions and various combinations of chromosomes 7 gain and 10 loss may be seen in glioblastoma, partial arm alterations are not considered sufficient by cIMPACT-NOW criteria at this time [29, 102]. Finally, *TERT* promoter mutations have a clear association with aggressive behavior in the setting of glioblastoma, and this is the most sensitive (66.7%) but least specific (89.4%) parameter, largely since *TERT* promoter mutations can be seen in other gliomas or systemic cancers [56, 57]. Critically, since *TERT* promoter mutations occur in a vast majority of molecularly defined (i.e. IDH-mutant and 1p/19q-codeleted) oligodendrogliomas, assessment of 1p/19q status and exclusion of an IDH mutation by sequencing is usually required before designating a molecular glioblastoma.

Another complicating factor is the presence of other genetic alterations which may preclude a molecular glioblastoma diagnosis because of their own prognostic implications. The presence of H3 K27M in an infiltrative astrocytoma is diagnostic of diffuse midline glioma, H3 K27-mutant, a separate molecularly defined entity. Similarly, the IDH-wildtype gliomas with histone H3.3 G34R/V mutation should be considered, especially in younger patients [96]. Emerging molecular entities such as diffuse astrocytomas with *MYB/MYBL* fusion, *FGFR1* or *FGFR3* alterations, or *BRAF* alterations are associated with a more indolent clinical course, but do not yet have their own diagnostic categorization and would appear as IDH-wildtype [106]. Recently, through DNA methylation profiling of histologically defined anaplastic pilocytic astrocytoma, Reinhardt et al defined the entity of anaplastic astrocytoma with piloid features, a category of IDH-wildtype astrocytoma with piloid morphology and frequent MAPK pathway alterations, loss of *CDKN2A/B*, and loss of ATRX. These tumors have a better outcome than IDH-wildtype glioblastoma and are molecularly distinct from conventional glioblastoma groups, as defined by DNA methylation profiling [107]. Finally, there are diffuse astrocytic gliomas which lack IDH mutation, *EGFR* amplification, *TERT* promoter mutation, and the + 7/− 10 chromosomal alteration. In the absence of an identifiable molecular driver, such cases may be diagnosed by histologic features and designated as “Not elsewhere classified”.

**Fig. 6:**
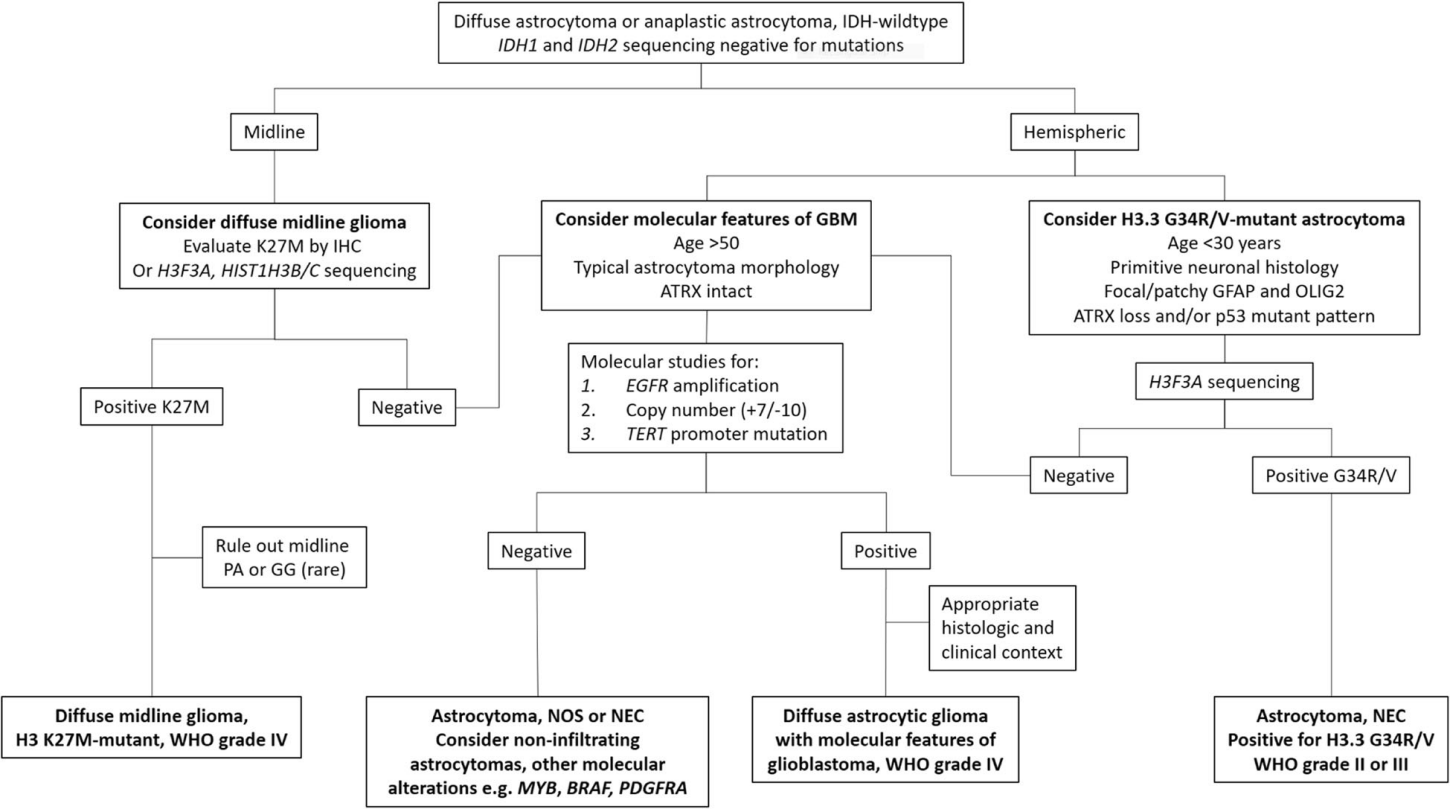
An approach to IDH-wildtype diffuse astrocytoma and anaplastic astrocytoma. See text for details. Abbreviations: IDH – isocitrate dehydrogenase; IHC – immunohistochemistry; ATRX – ataxia telangiectasia and mental retardation syndrome, X-linked; K27M – histone H3 K27M; G34R/V – histone H3.3 G34R or G34V; PA – pilocytic astrocytoma; GG – ganglioglioma; GBM – glioblastoma; NOS – not otherwise specified; NEC – not elsewhere classified

In summary, for these three alterations, their presence alone is not sufficient for the molecular glioblastoma diagnosis and does so only with the histologic exclusion of other molecular-pathologic entities that may mimic diffuse astrocytomas, such as oligodendroglioma, other genetically-defined gliomas, or focal areas of infiltration in pleomorphic xanthoastrocytoma or pilocytic astrocytoma. The lack of absolute specificity for most of the alterations mentioned in this section, and the complexity of interpreting molecular findings in context of the histology, underscores the need for periodic updates to the molecular classification system. One possible approach to identifying these cases is presented in Fig. 6. Within the context of a diffuse astrocytic neoplasm, based on the tumor age and location, H3-mutant tumors (either H3 K27M or H3.3 G34R/V) can be considered, along with molecular features of glioblastoma which, if identified, should be taken in the proper clinical, radiographic, and histologic context. An integrated diagnosis and designation as WHO grade IV explicitly informs clinicians of the prognostic implications of the key molecular findings.

## Conclusions

Brain tumor classification is increasingly rooted in the molecular drivers underlying different tumor entities. Currently, the absolute number of integrated diagnostic entities is low relative to the rapidly growing body of research on molecular analysis of brain tumors. However, incorporation of molecular features into the classification of diffuse gliomas means that due to their frequency, a majority of the primary intra-axial brain tumors now require some degree of molecular data for accurate and up-to-date classification. Additional molecularly defined entities and integrated diagnostic approaches are expected as the field evolves. As the number and scope of brain tumor-associated molecular alterations increases, academic medical centers are moving toward next-generation molecular diagnostics, allowing for detection of a broad spectrum of molecular alterations [104, 108–112]. Rich and useful data can still be obtained with careful morphologic analysis, judicious use of immunohistochemical stains, targeted sequencing, and fluorescence in situ hybridization, technologies which are accessible at most institutions and together can give adequate classification of most infiltrating gliomas. Pathologists should recognize that rare molecular entities exist, and that in some cases additional molecular testing may be indicated to detect clinically relevant alterations that are associated with aggressive behavior, such as molecular features of glioblastoma in histologically lower grade diffuse astrocytic gliomas. Molecular information now forms an essential part in neuropathology diagnosis, and the repertoire of molecular testing options will grow and evolve over time.

## Abbreviations

ALT: Alternative lengthening of telomeres
ATRT: Atypical teratoid rhabdoid tumor
ATRX: α-thalassemia/mental retardation syndrome X-linked protein
cIMPACT-NOW: Consortium to Inform Molecular and Practical Approaches to CNS Tumor Taxonomy – Not Official WHO
CNS: Central nervous system
DAXX: Death-domain associated protein
EGFR: Epidermal growth factor receptor
ETMR: Embryonal tumor with multilayered rosettes
ETS: E26 transformation-specific
EZH2: Enhancer of zest 2
FISH: Fluorescence in situ hybridization
GABPA: GA-binding protein, alpha subunit
GBM: Glioblastoma
G-CIMP: Glioma CpG island methylator phenotype
GFAP: Glial fibrillary acidic protein
GG: Ganglioglioma
H3 K27M: Histone H3 lysine 27 to methionine mutant protein
H3 K27me3: Lysine position 27 trimethylated histone H3
H3.3 G34R/G34V: Histone H3.3 guanine 34 to arginine or valine mutant protein
IDH: Isocitrate dehydrogenase
IDH1 R132H: Isocitrate dehydrogenase 1 arginine 132 to histidine mutant protein
IHC: Immunohistochemistry
ISN: International Society of Neuropathologists
LOH: Loss of heterozygosity
MAPK: Mitogen-activated protein kinase
MGMT: O^6^-methylguanine-DNA methyltransferase
NEC: Not elsewhere classified
NOS: Not otherwise specified
OLIG2: Oligodendrocyte transcription factor 2
PA: Pilocytic astrocytoma
SNP: Single nucleotide polymorphism
TERT: Telomerase reverse transcriptase protein
WHO 2016: WHO Classification of Tumours of the Central Nervous System, Revised 4th Edition
WHO: World Health Organization
